# Reading disorders and dyslexia

**DOI:** 10.1097/MOP.0000000000000411

**Published:** 2017-02-01

**Authors:** Charles Hulme, Margaret J. Snowling

**Affiliations:** a Division of Psychology and Language Sciences, University College London; b St John's College, Oxford, UK

**Keywords:** decoding, dyslexia, intervention, language impairment, reading, screening

## Abstract

**Purpose of review:**

We review current knowledge about the nature of reading development and disorders, distinguishing between the processes involved in learning to decode print, and the processes involved in reading comprehension.

**Recent findings:**

Children with decoding difficulties/dyslexia experience deficits in phoneme awareness, letter-sound knowledge and rapid automatized naming in the preschool years and beyond. These phonological/language difficulties appear to be proximal causes of the problems in learning to decode print in dyslexia. We review data from a prospective study of children at high risk of dyslexia to show that being at family risk of dyslexia is a primary risk factor for poor reading and children with persistent language difficulties at school entry are more likely to develop reading problems. Early oral language difficulties are strong predictors of later difficulties in reading comprehension.

**Summary:**

There are two distinct forms of reading disorder in children: dyslexia (a difficulty in learning to translate print into speech) and reading comprehension impairment. Both forms of reading problem appear to be predominantly caused by deficits in underlying oral language skills. Implications for screening and for the delivery of robust interventions for language and reading are discussed.

## INTRODUCTION

Learning to read is one of the key outcomes for early education and children who have reading difficulties often enter a downward spiral of low educational attainment and poor employment prospects with negative consequences for adult well-being. When we consider problems in learning to read, it is important to make a clear distinction between decoding (the accuracy or fluency of reading aloud) and comprehension (the adequacy of understanding text). Problems in learning to decode (developmental dyslexia) and problems in learning to comprehend text (reading comprehension impairment) are distinct forms of difficulty, both of which appear to depend principally upon impairments of oral language development. As we will outline below, Dyslexia is related to early problems in oral language development, with persisting problems in the development of speech-sound (phonological) skills being a particularly important obstacle to learning to decode print. In contrast, reading comprehension impairment depends critically upon broader oral language difficulties; particularly problems with understanding word meanings, and problems with grammatical skills. 

**Box 1:**
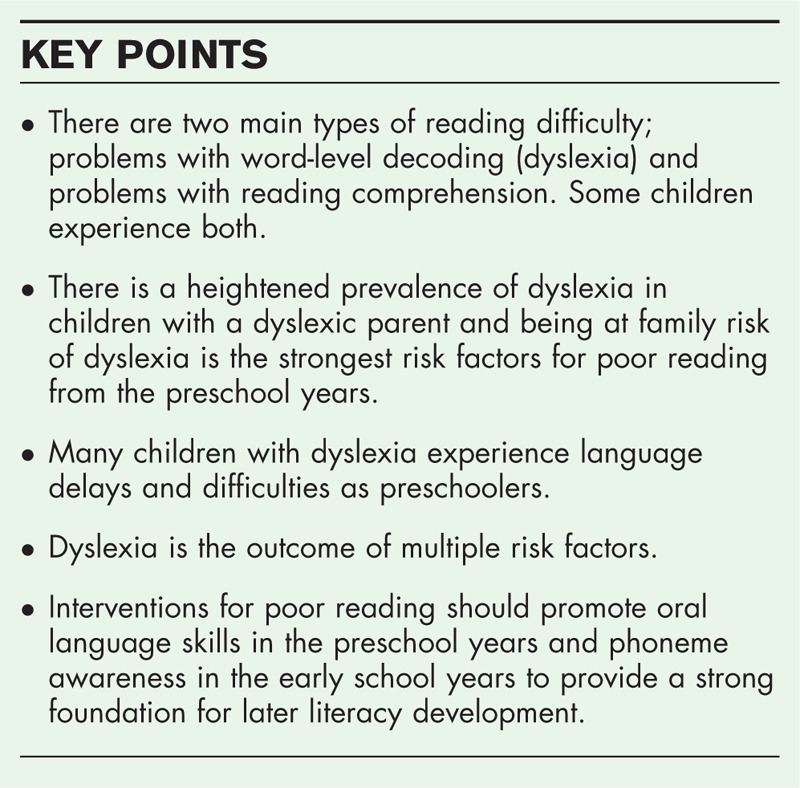
no caption available

## DYSLEXIA

### Definition and prevalence

Developmental dyslexia is the most widely used term for children who experience severe difficulties in learning to decode print. Children with dyslexia find it hard to recognize printed words, have great difficulties ‘sounding out’ unfamiliar words, and often also read slowly. In European languages, which have more regular writing systems than English, the main symptoms of dyslexia are poor reading fluency and spelling but the predictors of reading (and dyslexia) are the same, namely letter knowledge, phoneme awareness and rapid naming (RAN) skills [1].

It is important to emphasize that reading skills, like many other human characteristics (e.g., weight, blood pressure) show a continuous distribution in the population. As such, the criteria used for diagnosis are to some extent arbitrary (just as for obesity or hypertension). A common criterion for diagnosing dyslexia is reading accuracy more than 1.5 SD below the mean, which results in roughly 7% of the population being identified as dyslexic [2]. Dyslexia is more common in males, and is frequently comorbid with other developmental disorders such as specific language impairment (SLI), attention deficit hyperactivity disorder, or developmental coordination disorder [3].

### Risk factors for dyslexia

Dyslexia runs in families and twin studies have established that difficulties with reading and related skills are highly heritable. More recently, molecular genetic analyses have identified many potential candidate genes of small effect associated with individual differences in reading [4]. Notwithstanding the importance of genetic factors, the home literacy environment [5] and the quality of teaching are likely additional influences on reading development.

Furthermore, genes act through the environment and both passive and active gene–environment correlations can be expected to affect literacy outcomes. Parents with dyslexia not only share genes with their offspring, but also plausibly may provide a different home literacy environment to that found in homes where parents do not experience literacy difficulties. Similarly, children who are poor readers are less likely to seek out opportunities for reading than good readers and hence will have less exposure to print. Low levels of print exposure and reading practice will, in turn, compromise reading development.

For many years, studies of the likely cognitive causes of dyslexia were dominated by case–control studies of highly selected clinical groups. Such studies are subject to referral bias and often children with comorbid conditions are excluded from study samples. Arguably a more robust approach is offered by prospective longitudinal studies, which follow the development of children at high risk of dyslexia from the preschool years, to examine the characteristics of those who go on to be dyslexic to identify causal risk factors.

Studies of children at family risk of dyslexia by virtue of having a first degree affected relative have highlighted the crucial importance of language to literacy development. Several studies of children at family risk of dyslexia have been completed and others are ongoing [6^▪▪^]. These studies show a heightened prevalence of dyslexia in the offspring of affected parents, with some 44% developing dyslexia. These studies also show that dyslexia is not ‘all or none’. Rather, among children at family risk of dyslexia, literacy outcomes are distributed continuously with some children, while not qualifying for the label of dyslexia, nonetheless showing dyslexic symptoms, including relatively poor reading fluency and spelling.

The findings of family risk studies also show that single deficit accounts of dyslexia are inadequate. Although such studies confirm that poor phonological skills (e.g., poor nonword repetition in the preschool and poor phoneme awareness in the school years) are primary risk factors for poor reading, dyslexia is more likely to be ‘diagnosed’ when broader language impairments (including difficulties with grammar and poor vocabulary) persist into the school years. Such findings add to a growing body of evidence that a phonological deficit is one of a number of risk factors for dyslexia that accumulate towards a threshold [7].

## RELATIONSHIP BETWEEN DYSLEXIA AND LANGUAGE IMPAIRMENT

### Preschool profiles

It is well established that children with speech and language difficulties are at risk of literacy problems and there are likely to be shared endophenotypes between SLI, speech sound disorder, and dyslexia [8]. Bishop and Snowling [9] suggested that dyslexia and language impairment are both characterized by poor phonology (a shared risk factor for poor decoding) but differ in the extent to which broader language difficulties (vocabulary and comprehension) are implicated. However, few studies have made direct comparisons between the disorders as SLI tends to be diagnosed in the preschool years, whereas dyslexia is typically diagnosed much later (after a child has been in school for some years but failed to make adequate progress in learning to read).

Nash *et al*. [10], Hulme *et al*. [11^▪▪^], and Snowling *et al*. [12^▪^] followed the development of children at family risk of dyslexia and children with preschool language impairment in the Wellcome Language and Reading Project – a 5-year longitudinal study from age 3.5 to 8 years. Three groups of preschool children were recruited to the study: children at family risk of dyslexia, children considered to be language impaired, and children at low risk of dyslexia (controls). Following parental assessment of literacy skills, children were initially classified as either at family risk or not at risk. Next, following a language assessment, the children were grouped according to whether they fulfilled criteria for language impairment, defined as at least 1 SD below the mean on measures of receptive and expressive language skills. This procedure yielded four groups of children: typically developing, children with SLI, children at family risk of dyslexia (FR), and children at family risk of dyslexia with SLI. It is noteworthy that about a third of the children at family risk of dyslexia also had a preschool language impairment. These children did not differ from the SLI-only group on any phonological or language measure, whereas the FR-only group had difficulties only on phonological tasks that tapped speech production, namely articulation and word and nonword repetition. They also showed subtle and short-lived difficulties with grammar (problems with marking the past tense *– ed,* and third person singular *– s*) and in repeating sentences accurately. Gooch *et al*. [13] went on to show that the children in the SLI groups were more likely to have difficulties with attention and motor development than children at family risk without language impairment.

### Predictors of individual differences in reading

Hulme *et al.*[11^▪▪^] tracked the progress of the children from the three risk groups and controls at ages 3.5, 4.5, 5.5, and 8 years and used the data in a path model to investigate the causal relationships between language and literacy skills. At 3.5 years, a single language factor could be defined by performance on tests of vocabulary, grammar, conceptual understanding, repetition, and articulation (with repetition and articulation loading least well on this factor, possibly because they also tap speech processes). Language at 3.5 years predicted the foundations for decoding: namely letter knowledge, RAN, and phoneme awareness with the relationship between language and phoneme awareness being particularly strong. In turn, letter knowledge and phoneme awareness measured at 4.5 years predicted decoding skills at 5.5 years. Reading comprehension at 8 years of age was predicted by decoding at 5.5 together with language at 3.5 years.

These findings underline the importance of oral language development for reading development. The impact of language development at 3.5 years on decoding was fully mediated by phoneme awareness and letter knowledge, and there was a direct long range effect of language skills at 3.5 years on reading comprehension measured some 5 years later at age 8 years. A clear implication is that children with poor language skills in the preschool years are at high risk of developing both dyslexia and reading comprehension impairment. However, there is a complication. It is not uncommon for children who show delayed language development in the preschool years to ‘catch up’ and for speech difficulties to resolve. In this light, Bishop and Adams [14] proposed a ‘critical age hypothesis’ – only when children have problems with language (or speech) development that persist to the point of school entry will they succumb to reading problems.

### Trajectories of language development

To investigate language development in the Wellcome Language and Reading Project, and more specifically, to differentiate children with persistent versus resolving language impairments, Snowling *et al*. [12^▪^] examined the progress of individual children at three time points: preschool (3.5 years), school entry (5.5 years), and the middle of primary school (8 years). By assessing each child on tests tapping receptive and expressive grammar and vocabulary, it was possible to determine whether or not they fulfilled criteria for SLI at the different time points. In total, 66% of the children in the sample had normal language at all three times. Among the others, it was possible to discern three trajectories of development: 16% had language impairments that resolved, whereas 56% had persisting language impairments. In addition, some 28% showed late emerging language impairments; these children performed well within the normal range on language tasks at 3.5 years, yet scored as poorly as the persisting group at age 8 years. It is noteworthy that this ‘late emerging’ trajectory was strongly associated with familial dyslexia. In line with the critical age hypothesis, children with early language difficulties, which resolved by the start of formal literacy instruction tended to have good literacy outcomes, but children with both persisting and later emerging difficulties tended to have reading difficulties with more than 40% of each group affected.

### Screening and assessment

Given that dyslexia has its precursors in preschool, it may be possible to identify children early with a view to providing early intervention. However, screening is not straightforward because risk and protective factors interact during learning to read. Using data from a Finnish longitudinal study, Puolakanaho *et al.*[15] showed that being at family risk of dyslexia increases the probability of reading disability. However, if letter-naming skills develop early, the risk is considerably decreased. Similarly, for a child with poor letter-naming skills at 4.5 and 5.5 years, the probability of dyslexia is lower if he or she has good phonological awareness or efficient RAN.

In a similar vein, data from the Wellcome Language and Reading Project confirmed a changing pattern of prediction of dyslexia at different ages [16]. Although family-risk status was a stronger predictor of dyslexia from preschool than language difficulties were, at the time of school entry, poor language skill was a significant risk factor. Additional predictors in the preschool years included letter knowledge, phonological awareness, RAN, and executive skills and, at the time of school entry, motor skills slightly increased the prediction probability. These findings underline the fact that dyslexia is the outcome of multiple risk factors and confirm that children with language difficulties at school entry are at high risk. However, screening for dyslexia does not reach an acceptable clinical level until close to school entry when letter knowledge, phonological awareness, and RAN, rather than family risk, together provide good sensitivity and specificity as a screening battery.

## INTERVENTIONS FOR CHILDREN'S READING AND LANGUAGE DIFFICULTIES

We began by arguing that there are two types of reading disorder and we showed that the predictors of decoding (and hence dyslexia) differ from the predictors of reading comprehension. It follows that different forms of intervention are required for children with dyslexia compared with ‘poor comprehenders’. The evidence reviewed so far suggests that early interventions to strengthen the language foundations for reading will be important. There is growing evidence from randomized trials that educational interventions for reading and related learning disorders are effective when delivered by trained practitioners [17]. In general terms, interventions to promote decoding (and hence remediate dyslexia) comprise training in phoneme awareness linked to letter knowledge, together with systematic phonic instruction in the context of book reading. In contrast, interventions to promote reading comprehension involve work to improve oral language skills (including work on vocabulary and narrative skills) and emphasis on the use of inferences and metacognitive strategies to ensure coherent understanding of text [18].

Fricke *et al*. [19] evaluated an oral language programme aimed at improving vocabulary, grammar, narrative and active listening skills, and also assessed its impact on reading. Children receiving the intervention made greater gains in oral language and narrative skills than a waiting-list control group who received ‘business as usual’ and their phoneme awareness and letter knowledge also improved. Although the programme contained no activities directed towards reading, and no gains were made in decoding skills, an important outcome was that the intervention group showed significant gains in reading comprehension 6 months after the intervention had ended. Moreover, these gains were fully mediated by gains in oral language.

## CONCLUSION

Dyslexia represents the lower end of a continuous distribution of reading skills in the population. The oral language weaknesses that are the precursors of dyslexia can be observed in the preschool period. Being at family risk of dyslexia is an important risk factor and a major proximal cause is in speech processing (phonological) difficulties. Although dyslexia is primarily a decoding difficulty, many children also experience reading comprehension problems associated with co-occurring language difficulties. We believe that screening for language difficulties should be conducted at school entry to identify children who are at risk of difficulties in learning to read. There is good evidence from randomized trials that language interventions can ameliorate language weaknesses detected in the early school years. Similarly, specialist teaching methods are effective in ameliorating the problems in learning to decode print that are seen in children with dyslexia.

## Acknowledgements

None.

### Financial support and sponsorship

The study was funded by Wellcome Trust grant WT082032MA.

### Conflicts of interest

There are no conflicts of interest.

## REFERENCES AND RECOMMENDED READING

Papers of particular interest, published within the annual period of review, have been highlighted as:

▪ of special interest
▪▪ of outstanding interest
